# Improving patient-provider communication about chronic pain: development and feasibility testing of a shared decision-making tool

**DOI:** 10.1186/s12911-020-01279-8

**Published:** 2020-10-17

**Authors:** Nananda Col, Stephen Hull, Vicky Springmann, Long Ngo, Ernie Merritt, Susan Gold, Michael Sprintz, Noel Genova, Noah Nesin, Brenda Tierman, Frank Sanfilippo, Richard Entel, Lori Pbert

**Affiliations:** 1 University of New England and Shared Decision Making Resources, 1119 Five Islands Road, Georgetown, ME 04548 USA; 2 Northern Light Mercy Hospital, Portland, ME USA; 3 Beth Israel Deaconess Medical Center, Boston, MA USA; 4 Southern Maine Chronic Pain Support Group, Saco, ME USA; 5 Custom Communications, Portland, ME USA; 6 Sprintz Center for Pain and Dependency, The Woodlands, TX USA; 7 Penobscot Community Health Care, Bangor, ME USA; 8 Mainehealth, Portland, ME USA; 9 University of Massachusetts Medical School, Worcester, MA USA

## Abstract

**Background:**

Chronic pain has emerged as a disease in itself, affecting a growing number of people. Effective patient-provider communication is central to good pain management because pain can only be understood from the patient’s perspective. We aimed to develop a user-centered tool to improve patient-provider communication about chronic pain and assess its feasibility in real-world settings in preparation for further evaluation and distribution.

**Methods:**

To identify and prioritize patient treatment goals for chronic pain, strategies to improve patient-provider communication about chronic pain, and facilitate implementation of the tool, we conducted nominal group technique meetings and card sorting with patients with chronic pain and experienced providers (*n* = 12). These findings informed the design of the PainAPP tool. Usability and beta-testing with patients (*n* = 38) and their providers refined the tool and assessed its feasibility, acceptability, and preliminary impact.

**Results:**

Formative work revealed that patients felt neither respected nor trusted by their providers and focused on transforming providers’ negative attitudes towards them, whereas providers focused on gathering patient information. PainAPP incorporated areas prioritized by patients and providers: assessing patient treatment goals and preferences, functional abilities and pain, and providing patients tailored education and an overall summary that patients can share with providers.

Beta-testing involved 38 patients and their providers. Half of PainAPP users shared their summaries with their providers. Patients rated PainAPP highly in all areas. All users would recommend it to others with chronic pain; nearly all *trusted the information* and said it helped them *think about my treatment goals* (94%), *understand my chronic pain* (82%), *make the most of my next doctor’s visit* (82%), and *not want to use opioids* (73%)*.* Beta-testing revealed challenges delivering the tool and summary report to patients and providers in a timely manner and obtaining provider feedback.

**Conclusions:**

PainAPP appears feasible for use, but further adaptation and testing is needed to assess its impact on patients and providers.

**Trial registration:**

This study was approved by the University of New England Independent Review Board for the Protection of Human Subjects in Research (012616–019) and was registered with ClinicalTrials.gov (protocol ID: NCT03425266) prior to enrollment. The trial was prospectively registered and was approved on February 7, 2018.

## Background

Chronic pain is one of the most common reasons adults seek medical care [1]. Opioids became the mainstay of chronic pain treatment in recent decades. When properly used, opioid therapy can improve patients’ quality of life, decrease healthcare costs, and improve work productivity. However, misuse and abuse of prescription opioids has reached epidemic proportions (http://www.fda.gov/Drugs/DrugSafety/InformationbyDrugClass/ucm330614.htm). The number of drug overdose deaths increased 4-fold between 1999 and 2018 [2, 3], with opioid-related deaths accounting for two-thirds of all drug overdose deaths in 2016 and 2017 [4, 5]. Factors that drove the overuse of opioids were misinformation about its benefits and harms [6], inadequate training of health care providers in pain management, too little clinic time spent with complex patients, and too few multidisciplinary treatment programs [7, 8]. Yet pain remains undertreated among those most in need. Today’s prescription drug crisis reflects a broad failure in managing pain.

Lacking objective measures of pain, healthcare providers must trust their patients’ words to understand their pain, its impact on their lives, and their response to treatments. But pain and its treatments can interfere with patients’ ability to communicate [9]. Because pain can be exaggerated for secondary gain (for example, liability tort claims) or to obtain opioids that can be misused [10, 11], suspicion may be cast on patients with chronic pain [12]. Differences between patients’ and providers’ attributions about causes of pain further erode the trust that is essential to good communication. Patients who believe their pain is biologic may feel misunderstood when psychological treatments are recommended, or frustrated when no *diagnosis is identified* [13]*.* Mandated restrictions to treatments (such as dosing limits) may be blamed on providers, reinforcing feelings of distrust [14]. It is not surprising that many patients with chronic pain feel stigmatized and misunderstood [15] and report being labeled as hypochondriacs or drug seekers [16], while providers feel frustrated and overwhelmed [17, 18].

Helping patients communicate their experiences and beliefs about their pain, expectations about treatments, and treatment goals should help providers understand their patients better, which in turn should improve the effectiveness of pain management. The quality of patient–provider communication predicts patient satisfaction better than decreases in reported pain [19–22]. Understanding a patient’s goals, values, and expectations is essential because these elements serve as filters through which patients screen their options [23], interpret evidence [24], select treatment [25, 26], and respond to treatment [27]. Patients’ and providers’ treatment goals [28] and preferences often diverge [29, 30], reinforcing the need for providers to communicate with patients about their goals.

Providers can encourage or impede patient involvement [31]. Providers who engage in partnership-building and supportive talk create opportunities for patients to discuss their needs and be involved [32]. Yet providers only occasionally use partnering or supportive communication, inform patients when there is a decision to be made, present all treatment options, or encourage patients to consider their preferences and goals to guide treatment decisions [33, 34]. Patients who ask questions and express their concerns and preferences elicit more information and support from their provider [35–37], but many patients are reluctant to engage in collaborative discussions with providers for fear of being seen as a “difficult patient” [38]. Simply asking patients for their preferences is unlikely to yield meaningful responses because patients often have difficulty applying their values to health decisions [33, 34]. Because most providers are not taught how to engage patients in decisions about their health, clinical tools are needed to help with these tasks.

Shared decision-making (SDM) tools, such as decision aids, have been shown in clinical trials to help patients be more informed, experience less decisional conflict, be more involved in decisions about their health, and be more likely to choose treatments that are consistent with their informed preferences and values [39]. However, decision aids for chronic musculoskeletal pain appear far less effective than those developed for other conditions, improving only patients’ knowledge about treatment options [40]. A recent systematic review identified 17 randomized controlled trials of decision aids for chronic musculoskeletal pain, all targeting specific pain etiologies (such as hip or knee osteoarthritis). Only four decision aids addressed conservative management strategies [41–44]. Most [12] focused on decisions to undergo surgery. These tools have limited relevance to people with chronic pain for whom surgical intervention may no longer be a viable option and whose pain is often multifactorial in etiology, requiring multimodal treatment approaches.

Utilization of decision support tools in general has been disappointingly low, perhaps because they are typically designed with limited input from patients, little attention to factors that drive utilization, and are uninformed by theory [45]. Patient input, when elicited, is typically achieved through focus groups, but extracting patient priorities from these discussions is subjective and influenced by the investigator’s perspectives [46]. In contrast to traditional focus groups, the nominal group technique (NGT) coupled with card sorting, referred to as cognitive mapping, minimizes investigator bias, captures the language of each participant, maintains the autonomy of individual viewpoints, allows equal input by each participant, and objectively prioritizes and organizes findings [47–49]. User-centered design has been proposed as a way to make PDAs more suitable for clinical implementation [50].

To improve utilization, we worked directly with patients and providers, employing user-centered design methods that minimize investigator bias to design the tool.

International standards for developing decision aids [51] recommend that the development process follow a systematic process that involves consultation with patients and clinicians. The process should include scoping and design, developing a prototype, iterative ‘alpha’ testing with patients and clinicians, and ‘beta’ testing in ‘real life’ conditions before producing a final version for use and/or further evaluation. However, only about half of the published reports on decision aids included in the Cochrane review of treatment and screening decision aids [52] were field tested with patients and even fewer had been reviewed or tested by clinicians not involved in the development process. Additionally, few described how they reviewed and synthesized clinical evidence or a distribution strategy.

In order to improve the management of chronic pain, we aimed to develop a user-centered tool that improves patient-provider communication about chronic pain. This manuscript describes findings from cognitive mapping, the development of the tool, and patient and provider responses to the tool.

## Methods

### Beta-testing study: participants and recruitment strategy

We limited participants to English-speaking adults with access to the Internet who had a diagnosis of a chronic musculoskeletal or neurologic pain disorder resulting in persistent pain lasting over 6 months. We excluded patients whose pain was primarily gastrointestinal, genitourinary, or cardiac in origin because these patients represent a separate medical entity that requires treatment of the underlying cause. We limited beta-testing participants to those with an upcoming appointment to manage their chronic pain within 8 weeks. We included providers who were doctors of medicine (MD), doctors of osteopathic medicine (DO), physician assistants (PA), nurse practitioners (NP), and social workers (MSW) providing they had 2 or more years of clinical experience managing 10 or more patients per month with chronic pain. To ensure that only subjects with a diagnosis of chronic pain were included, participants could only be referred by patient advisers or participating providers who had access to chronic pain patients or patient networks. We purposefully selected diverse patients from different regions of the country. Patient referral networks, including the American Chronic Pain Association), the Southern Maine Chronic Pain Support Group, and a network of multiple sclerosis patients with chronic pain [53] distributed email invitations to their members. We limited beta-testing referrals to a convenience sample of providers who manage patients with chronic pain in Maine and Texas, including pain management centers, primary care settings, and addiction and mental health clinics. We sent participating providers referral cards and emails to distribute to potentially eligible patients. Beta-testing recruitment occurred between November 15, 2017, and June 4, 2018.

Formative research using cognitive mapping informed the design of the PainAPP tool. We conducted structured focus groups using the NGT to identify i) patient treatment goals and preferences, ii) strategies to improve the clinical dialogue surrounding chronic pain, and iii) implementation strategies for distributing the tool, using previously validated methods [53, 54].

NGT groups of 5–9 patients or providers responded to one of the following questions: *What are the things you want to accomplish in treating your pain?* (only patients); *What would make it easier to communicate with your doctor [patients] about chronic pain and how to manage it?* (patients and providers); and *What are the top features that we need to design into the tool to make it both practical and helpful to you in managing your patients with chronic pain?* (only providers).

**Fig. 1:**
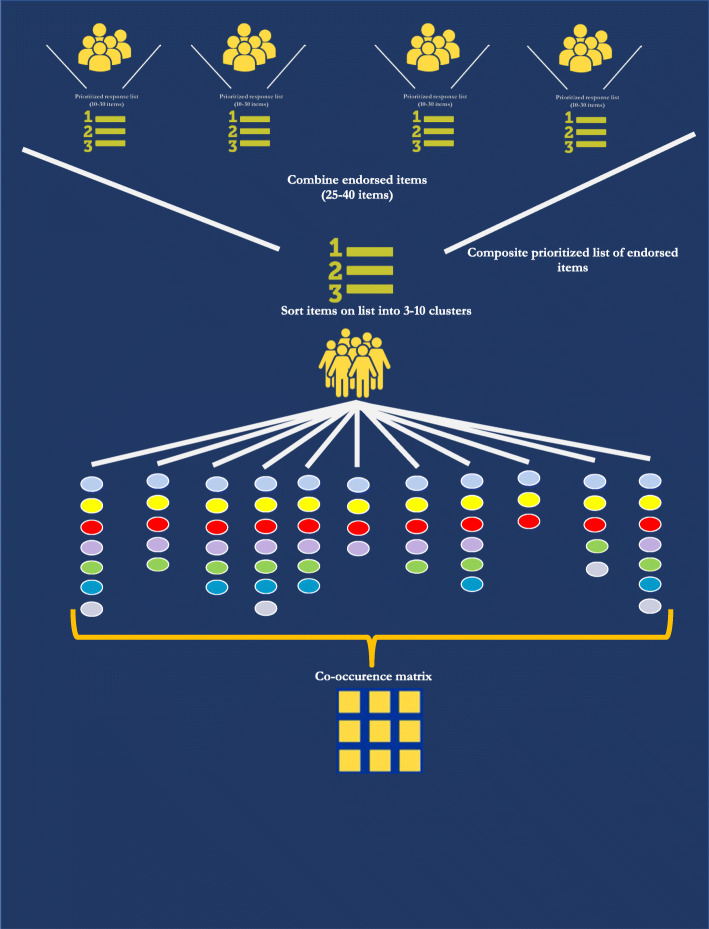
Overview of cognitive mapping methods used for PainAPP

NGT participants silently wrote down their responses, which were later shared, consolidated, and ranked by participants. We later conducted card sorting among a larger sample to organize treatment goals into meaningful clusters. We used hierarchical cluster analysis and multidimensional scaling [55–58] to construct a visual representation (cognitive map) of the data and identify conceptual domains based on how frequently items were sorted into the same category. Figure 1 outlines these methods. All analyses were performed using SPSS Statistics 23.

The content in PainAPP was guided by findings from this formative work, relevant theory (Table 1) [59, 60], and SDM guidelines [61]. PainAPP includes the key elements identified by patients and providers during cognitive mapping: assessing patient treatment goals and preferences, functional abilities, and pain; providing tailored education; and generating a summary that patients can share with their providers. To elucidate topics identified by patients and providers, we created short unscripted videos of people discussing their actual experiences with chronic pain. We also created a short motion graphics video to introduce the tool and explain its use and navigation [62]. All patient-facing content was co-written and iteratively revised by people with chronic pain and reviewed by experienced providers for scientific accuracy. Usability and beta-testing among patients further guided the content and design of PainAPP, resulting in substantial shortening and redesign, including splitting the tool into 2 linked parts.

**Table 1:** Theory-based features of PainAPP

Theory-based recommendation^**Error! Bookmark not defined.**^	Feature in tool
Optimize representation	All preference items and content were derived from and organized by experienced patients
Include all potentially appropriate options and their attributes	All relevant attributes of all options are shown to patients
Suspend selection of an initially favored option (pre-selection)	Start by focusing only on attributes, not options. Introduce options afterwards
Remind decision maker of the array of values	Includes activities that require attention to the complete array of values (broad and narrow): choosing, ranking, rating
Facilitate weighting of attributes	Force selection of top 3, then ranking, then rating of each subcomponent.

### Beta-testing protocol

We emailed a link to the study website to patients who responded to the study invitation. The link directed them though the eligibility screener, informed consent document, and baseline questions. Patients were then randomized to either PainAPP or the control group (using Qualtrics’ randomization feature). Baseline questions were tailored to the assigned study group to avoid duplication of items that would be subsequently asked in PainAPP. Subjects randomized to PainAPP were emailed a unique, nontransferable link to the PainAPP tool; control subjects were directed to the control website (https://www.theacpa.org/pain-management-tools/communication-tools/). The control website was chosen based on recommendations of advisers and published quality ratings [63]. Both study groups were emailed identical evaluations immediately after viewing the assigned intervention, after their scheduled provider appointment, and 3–4 weeks later. Patient participants received an incentive payment ($25 online gift card) after completing the first evaluation and a $5 gift card for each subsequent evaluation; providers received a single $50 gift card.

### Beta-testing evaluation and measures

We used a randomized controlled prospective study design, using an initial 4:1 assignment to PainAPP versus control to be able to assess uptake and utilization of PainApp so that any necessary changes could be addressed. Evaluations obtained after viewing the assigned intervention included: “I would recommend it to others with chronic pain,” “I trusted the information provided,” “It helped me think about my treatment goals,” “It presented unbiased information,” “It addressed topics that are important in communicating with my doctor,” “It helped me manage my chronic pain,” “It will help me make the most of my doctor’s visit,” “It helped me think about the pros and cons of opioids,” “It changed the way I think about opioids,” and “It made me **not** want to use opioid pain medications.” Survey responses used a 5-point Likert scale (“strongly agree, somewhat agree, neither agree nor disagree, somewhat disagree, strongly disagree”). Participants were also asked for their suggestions for improving PainAPP.

The following measures were assessed after the subjects’ scheduled provider appointment:

*Interest in sharing their summary report with their provider*. Subjects were asked: “If you received a personal summary report, did you try to share your personal summary report with this provider?” Responses were “yes,” “no,” “not sure,” and “not applicable—I did not receive a personal summary.”

*Patient-provider communication*. Items from the Consumer Assessment of Healthcare Providers and Systems (CAHPS) Adult, Clinician & Group Survey [64] that were related to SDM were summed to create a composite measure.

*Satisfaction with communication* was assessed by using the 10-item subscale of the.

The Combined Outcome Measure for Risk Communication and Treatment Decision Making Effectiveness (COMRADE) [65]. Scores are summed to produce a total score (0–20). A higher score corresponds to higher satisfaction.

*Pain Intensity* was assessed using The Brief Pain Inventory, which rates the severity of pain at its worst, least, average, and currently, and the degree to which it interferes with daily activities (walking, work, mood, enjoyment of life, relations with others, and sleep).

The *Control Preference Scale* assesses “the degree of control an individual wants to assume when decisions are being made about medical treatment,” with roles including the patient making treatment decisions alone, jointly with the physician, or the provider making decisions. Collaborative roles are generally considered preferable to having the provider or patient make decisions alone.

*Attitudes towards opioids:* For people currently using an opioid, we adapted a survey item related to intention to stop using or reduce opioids from the Prescribed Opioids Difficulty Scale [66]. For those not currently using an opioid, we used the questions: “It made me not want to use an opioid,” “It helped me think about the pros and cons of opioids,” and “It changed the way I think about opioids.”

### Beta-testing data analysis

All data were directly entered by the patient into a Qualtrics database. Use of PainAPP pages was monitored via analysis of website log files. Mean time spent on different activities was computed after removing outliers that likely reflected break periods. We could not directly determine how much time participants spent on the control website because we did not have access to that website’s log file. Because of the small sample size, the limited scope of the beta-testing, and biases detected during beta-testing, limited statistical analyses were performed. Recruitment was ended before our initial target of 50 patients was achieved because of serious challenges discovered that prompted a reassessment of the study design. The initial target sample of 50 was estimated to provide an 80% chance of detecting a 20% improvement on the primary outcome COMRADE with a type-I error rate of 0.05 [67].

### The intervention

**Fig. 2:**
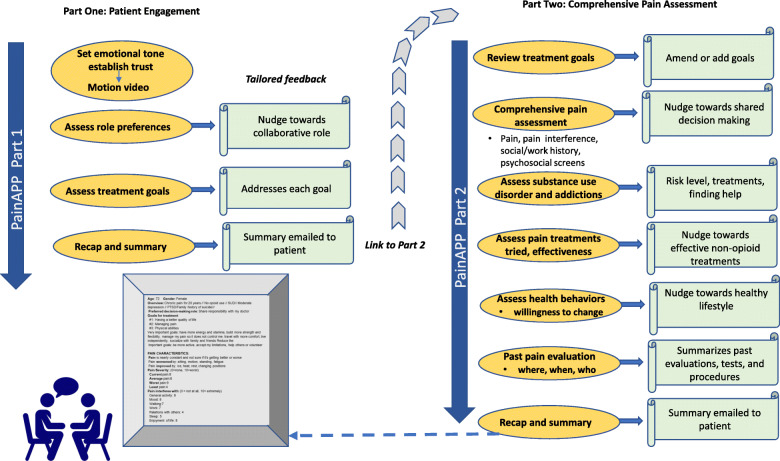
Content diagram of PainAPP

**Fig. 3:**
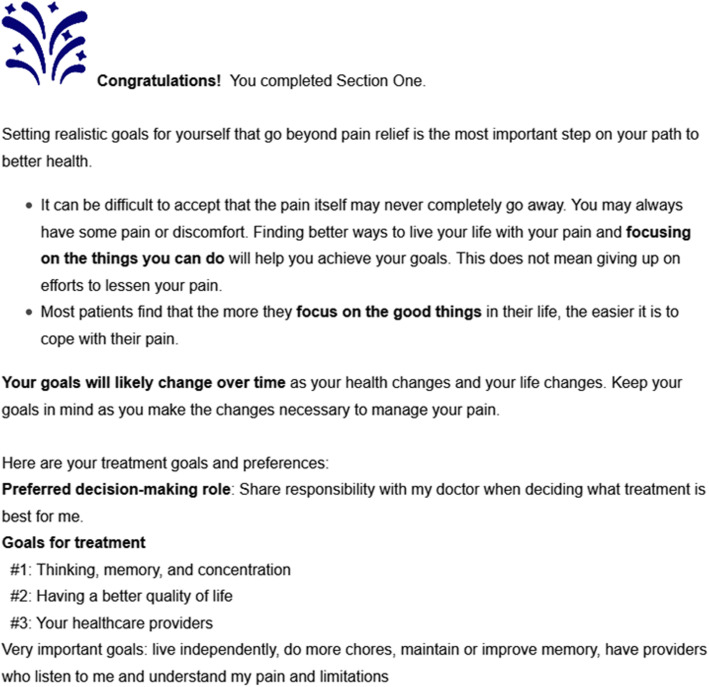
Sample summary of patient treatment goals and preference

PainAPP is an interactive, online decision support tool that employs a tunnel design [68] to guide users through a series of structured, interactive modules (Fig. 2). Multi-tiered values clarification exercises help users identify and prioritize their treatment goals and preferences by asking them to select and rank the most important broad goals from a list of nine domains (for example, “managing pain,” “having a better quality of life,” and “reducing fear, anger, and depression”). Next, users are asked to rate the importance of more detailed goals that are conceptually related to the goals that were selected, using a 4-point Likert scale (“not important or does not apply,” “somewhat important,” “important,” and “very important”). All pre-programmed treatment goals were generated by patients with chronic pain during cognitive mapping. Additional goals can be added by the user. A summary of the patient’s goals and preferences is generated along with guidance on using these goals to manage pain (Fig. 3).

**Fig. 4:**
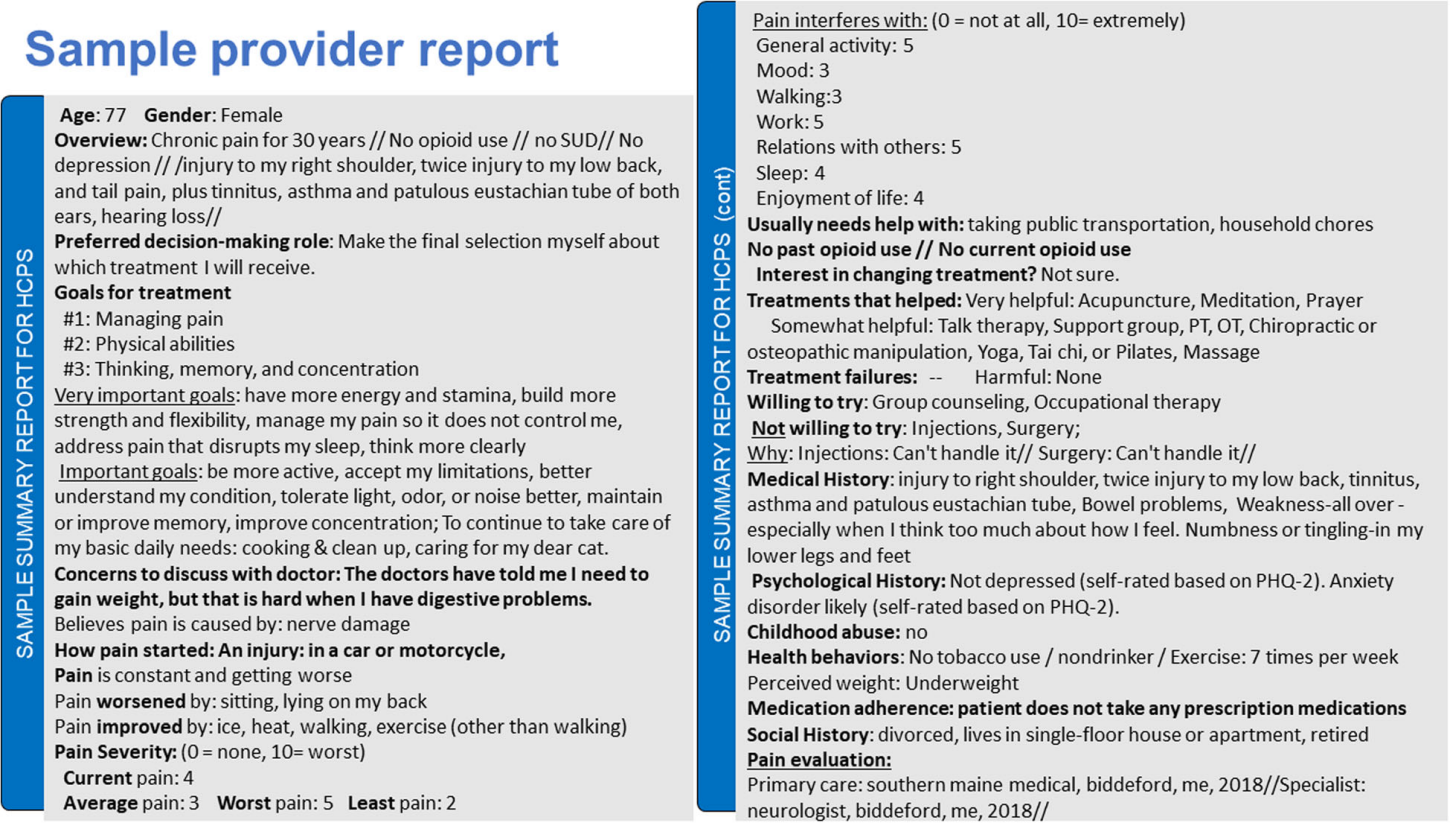
Sample provider summary generated by PainAPP

PainAPP consists of 2 linked parts that can be used independently or sequentially. Part 1 focuses on patient engagement, clarifying patient goals and preferences and providing tailored education. Part 2 includes a comprehensive pain assessment (including risk for opioid misuse, psychosocial comorbidities, and lifestyle risk factors) coupled with educational feedback tailored to the patient’s reported risks and preferences. Information entered in Part 1 is recalled in Part 2. Both parts generate a succinct structured summary, shown during use and emailed to the patient upon completion (Fig. 4). The patient can share the summary with his or her provider by either copying it into the patient portal, emailing it, printing it, or through a mobile device.

The tool emphasizes patient engagement, provider partnership, and communication. Features include: accessibility on multiple platforms, scalability, encryption, HIPAA compliance, and various options for dissemination. Content employs principles of effective communication [34] such as positive framing, side-by-side comparisons, graphics, encouragements and commendations upon completing sections, plain language, highlighting of important information, and a user-driven path. The tool was created using customized Qualtrics© software (Seattle, WA). The study adheres to CONSORT guidelines.

## Results

### Part 1. Cognitive mapping

#### Treatment goals

Sixteen patient subjects consented (50% female, 87% white, mean age 57 years) and completed the NGT activities. Two NGTs involving 8 patients each, conducted in February and April 2016, yielded 37 unique patient-identified treatment goals. Sixteen patient subjects consented to the card sorting exercise and completed all ratings; 15 completed the card sorting (94%). The 2-dimensional solution for the multidimensional scaling analyses indicated robust goodness-of-fit measures (stress = .1041). The most endorsed goal for patients (100%) was “To have providers who understand my pain and limitations” (Table 2). Ten patient clusters were derived from the 37 patient treatment goals. The most important cluster was *Physical activity and stamina*, followed by *You and your providers*, and *Pain management*. The least important was *Medication management*.

**Table 2:** Ratings of prioritized patient treatment goals (“Topics most important to you when you are choosing ways to manage or treat your chronic pain*”)*

#	Goal	Importance Rating (Mean, SD)	
**Physical activity and stamina**		**9.71**	**0.48**
To be able to do more with less pain		9.79	0.41
To have more energy and stamina and less fatigue		9.79	0.56
To pace myself, set reasonable expectations, listen to my body, and know my limitations		9.71	0.45
To be more physically fit (strength and flexibility) within my limitations		9.57	0.49
**You and Your Providers**		**9.62**	**0.50**
To have providers who understand my pain and limitations		10.00	0.00
To have a team of providers who work together to help me		9.71	0.59
To be able to explain better to my providers how I feel		9.14	0.91
**Pain Management**		**9.45**	**0.62**
To reach a point where pain does not control my day		9.79	0.41
To manage pain at a reasonable level		9.50	0.50
To learn more about chronic pain or my condition and how to deal with it better		9.29	0.80
To learn strategies to help me ease my pain		9.21	0.77
To be able to tolerate things like light, odor, noise		8.21	1.15
To reduce pain at night [allowing me to sleep better]		9.07	1.03
**Costs**		**9.29**	**0.80**
To find a treatment [or medical equipment I can afford]		9.29	0.80
To find an alternative treatment I can afford			
**Living a more independent and satisfying life**		**9.23**	**0.80**
To travel or commute with more comfort		9.36	0.61
To be able to sit comfortably		9.36	0.81
To be able to live independently in my home		9.21	1.01
To be able to do more household chores		9.00	0.76
To help others		9.57	0.49
To lead a fuller life		9.57	0.62
To be able to work		8.43	1.05
**Better memory, thinking, and focus**		**9.07**	**0.88**
To improve or maintain clear thinking		9.07	0.88
To retain and recall information			
To be better able to focus			
**Managing depression and emotions**		**8.91**	**0.91**
To be less irritable and able to deal better with stress		9.29	0.80
To be able to look forward to the future instead of being focused on my pain		9.21	1.01
To develop better ways to deal with depression		9.00	1.00
To be able to control anger and aggressive thoughts		8.57	0.90
To reduce my fear of being in pain		8.50	0.82
**Friends, family, and intimacy**		**8.88**	**1.08**
To maintain relationships with family and friends		9.07	0.96
To be able to socialize and reduce isolation		8.86	0.99
To improve or maintain sexual relations		8.71	1.28
**Minimal and natural treatments**		**8.77**	**0.76**
To avoid side-effects that cause more problems		9.57	0.49
To avoid surgery		9.29	0.80
To be able to handle pain using natural and/or alternative treatments		9.14	0.64
**Medication management**		**8.52**	**1.12**
To minimize or eliminate my pain medications		8.71	1.10
To maintain current level of pain medications		8.29	1.03
To have enough medication to adequately treat my pain		8.57	1.24
To become pain-free regardless of side effects		7.07	1.10

**Fig. 5:**
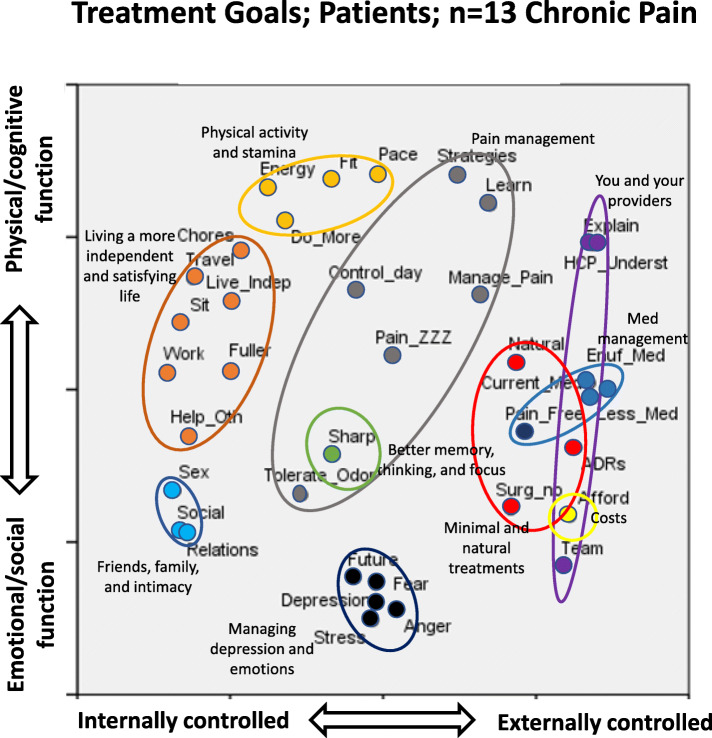
Cognitive map of patient treatment goals (*n* = 13)

We interpreted one dimension of the patients’ cognitive map as ranging from factors internally controlled by the patient to factors externally controlled, the other dimension ranging from emotional/social function to physical/cognitive impact (Fig. 5).

#### Communication strategies

Three NGTs involved 14 patient participants (67% female) and 7 provider participants (MD, DO, and PA). Forty-one unique communication strategies were generated by patients, 35 by providers. The most important patient-generated strategies were: “clinician takes me seriously and respects my input” and “being involved as an integral part of my treatment team.” The most important provider-generated strategy was “knowledge of past work-up,” followed by “timeline of the pain.” Strategies prioritized by both patients and providers were “improved provider teamwork,” “more time allotted for consultations,” and “better knowledge about the underlying condition.” Both groups prioritized knowledge about their condition over knowledge about treatment options (Table 3).

**Table 3:** Comparison of patient and provider identified communication strategies to improve chronic pain discussions

Top 10 patient factors	Top 10 providers factors
The clinician takes me seriously and respects my input	Past work-up and outcomes of the presenting problem/ source of pain
Be told about all options and side- effects	Having a clear timeline of the pain
The clinician is knowledgeable about my particular condition	Hearing how they believe their pain has impacted their daily lives, outlook, & relationships
Ask how **I** want to manage it/ accept that I am in charge of my health/ I am involved as an integral part of my treatment team	Listen to their story, allow them to vent
Take the time needed, not rush	Hear the patient's expectations about their pain
Talk to me not the computer/ make eye contact/ read the medical file before, not during, the appointment	A clear history of past treatments tried and whether they helped or not
Let me know that he’s communicated with other docs taking care of me	Have enough time with the patient (>30 minutes)
To have an encouraging doctor/ never leave me feeling utterly helpless, hopeless, or written off	Knowing of any adverse childhood experiences
Have a trusting relationship with my provider	Better use of behavioral and mental health
No judgment/ not look at me as if I’m faking it/ feel like doctors aren't stigmatizing me for my pain or way of life	Clarify common misunderstandings about pain* Assess patient’s support system*

*signifies that these 2 items were tied for 10th place

#### Clinical integration

We conducted one NGT involving 5 provider participants. Providers felt the tool would be most helpful if it helped them assess functional abilities and pain history (Table 4).

**Table 4:** Top ranked features to improve clinical integration **(**providers only**)**

Rank	What are the top features that we need to design into the tool to make it both practical and helpful to you in managing your patients with chronic pain?
1	Assess functional abilities and limitations
2	Provide pain history components (location, duration, history of onset, history of evaluation to date, associated symptoms, aggravating and ameliorating factors, current treatment, tried but failed treatment, etc)
3	Presenting a patient prioritized list of the patient’s goals for the encounter
4	Educate the patient on effectiveness of CBT, ACT and the emotional components of pain
5	Scripts to guide difficult conversations regarding changing a longstanding treatment plan
6	Educate the patient that reporting pain will not lead to prescription for pain med.
7	Screening tools for substance use disorder and/or opioid use disorder
8	Resource list customized by community (treating substance use disorder, acupuncturists, osteopaths, chiropractors, massage therapists, Tai Chi, etc.)
9	Require minimal effort by providers (not have to click too many boxes or write a whole lot)
10	Easy to read and follow
11	Facilitate the patient providing a signed release to facilitate obtaining past records in advance of the patient encounter
12	Keep it short
13	Gives multiple choice options which reflect the wide range of patient/provider possible responses
14	Include tools to help with motivational interviewing regarding pain and function
15	Easy access to MME calculator

### Part 2. Beta-testing findings

**Fig. 6:**
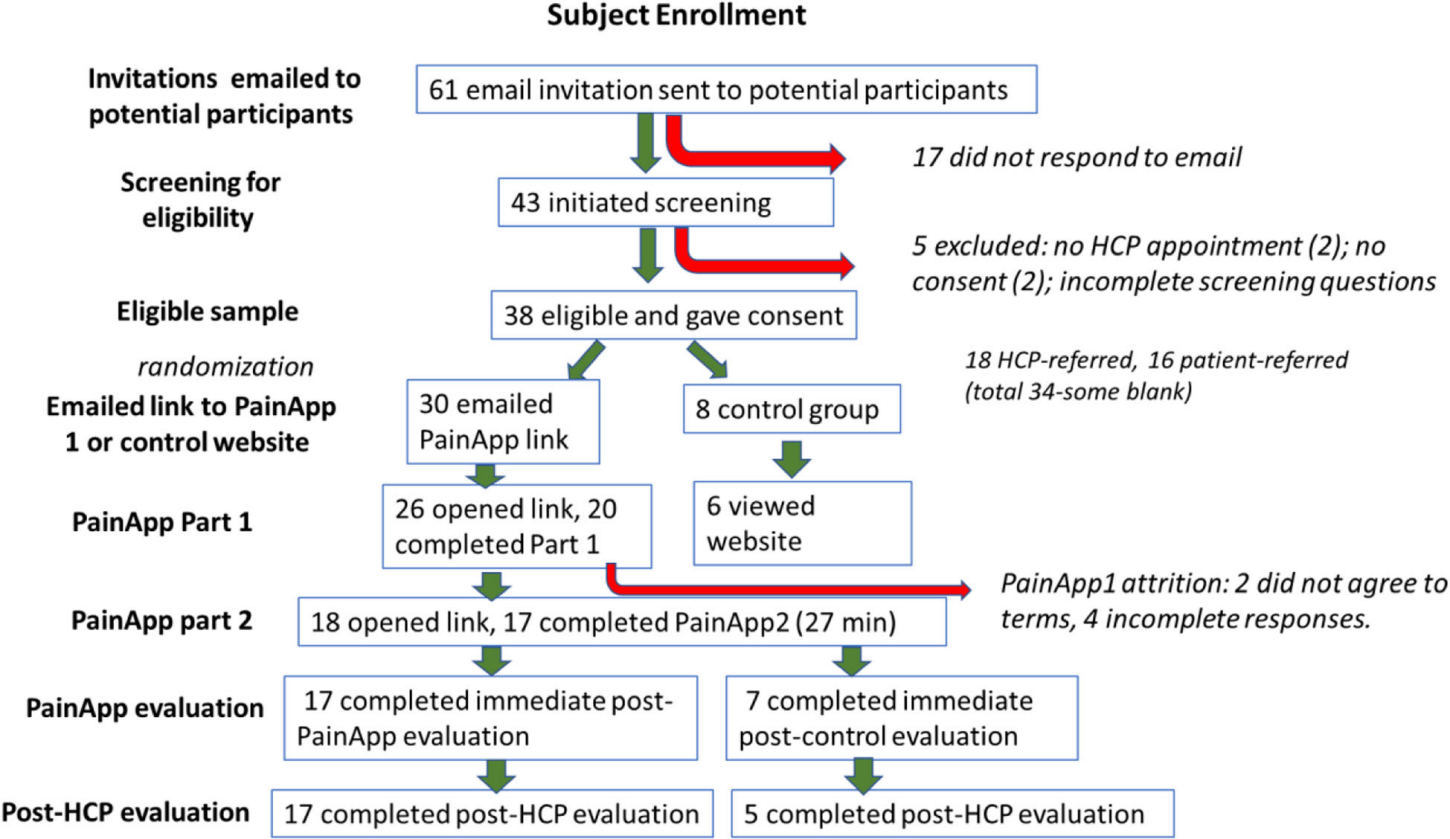
Overview of subject enrollment for beta-testing

Of the 61 patients who opted in and were emailed invitations to participate, 43 (70%) initiated screening, and of those, 38 (88%) were eligible and consented (Fig. 6). Completing the intake process took 20.7 min on average; *Patient Engagement and Education* (Part 1) took an average of 28.0 min; *Comprehensive Pain Assessment* (Part 2) took an average of 27.3 min. Patients using the control site reported spending between 15 and 60 min on the site (average 28 min). Just over half of patients (56%) were referred by providers, the remainder (44%) by patient networks. Socio-demographics of participants are shown in Table 5.

**Table 5:** Baseline patient participant demographic and clinical characteristics (beta-study) (*n* = 38)

Characteristic	Combined (n = 38)	PainAPP (*n* = 30)	Control (*n* = 8)
**Mean age**, years (range)	56.5 (22–80)	56.9 (22–75)	55 (41–80)
**Gender,** n (%)			
Male	12 (31.6)	9 (30.0)	3 (37.5)
Female	26 (68.4)	21 (70.0)	5 (62.5)
**Race**, n (%)			
White/Caucasian	32 (84.2)	25 (83.3)	7 (87.5)
Black or African American	2 (5.3)	2 (6.7)	0
Latino or Hispanic	2 (5.3)	1 (3.3)	1 (12.5)
Native American or Alaska Native	2 (5.3)	2 (6.7)	0
Other	0	0	0
**Education**, n (%)			
High school graduate or GED	6 (15.8)	5 (16.7)	1 (12.5)
Some college or 2-year college or technical school	10 (26.3)	7 (23.3)	3 (37.5)
4-year college graduate	10 (26.3)	7 (23.3)	3 (37.5)
More than 4-year college degree	12 (31.6)	11 (36.7)	1 (12.5)
**Primary cause of chronic pain** (%)			
Back pain	14 (36.8)	13 (43.3)	1 (12.5)
Fibromyalgia	6 (15.8)	4 (13.3)	2 (25.0)
Neck pain	4 (10.5)	4 (13,3)	0
Nerve pain	4 (10.5)	3 (10.0)	1 (12.5)
Other musculo-skeletal	8 (21.1)	5 (16.7)	3 (37.5)
Complex Regional Pain Syndrome	1 (2.6)	0	1 (12.5)
Other	1 (2.6)	1 (3.3)	0
Years with chronic pain, Mean (SD; range)	17.53 (12.13; 2–50)	17.45 (13.0; 2–50)	16.13 (8.2; 8–30
**Past alcohol or drug problem,** n (%)	6 (15.8)	5 (16.7)	1 (12.5)
Current daily tobacco use	8 (21.1)	7 (23.3)	1 (12.5)
**Use of opioid medications**, n (%)			
Yes, currently	17 (44.7)	13 (43.3)	4 (50.0)
Yes, in past but not now	12 (31.6)	10 (33.3)	2 (25.0)
No	9 (23.7)	7 (23.3)	2 (25.0)
**Overall health** (self-reported)			
Excellent	0	0	0
Very good	11 (9.0)	9 (30.0)	2 (25.0)
Good	15 (39.5)	12 (40.0)	3 (37.5)
Fair	12 (31.6)	9 (30.0)	3 (37.5)
Poor	0	0	0
**Overall mental or emotional health** (self-reported)			
Excellent	4 (10.5)	4 (13.3)	0
Very good	9 (23.7)	6 (20.0)	3 (37.5)
Good	13 (34.2)	13 (43.3)	0
Fair	8 (21.1)	5 (16.7)	3 (37.5)
Poor	4 (10.5)	2 (6.7)	2 (25.0)

Of the 17 patients who completed both components of PainAPP, half [7] wanted to share their summary report with their provider; 3 (37.5%) by printing it and bringing it to the clinic visit, 3 (37.5%) requested that it be mailed to them because they do not have a printer, and 2 (25%) opted to use a patient portal. Among the 16 subjects assigned to PainAPP who completed the post-provider visit, half [7] reported trying to share their summary report with their provider, 2 weren’t sure, 2 reported that it was not applicable because they did not receive it, and 4 did not try to share it. Among the 4 who reported not sharing their summary, 1 forgot, 1 reported that there was “no time and [their provider] did not seem that accessible”; one gave a non-informative response^1^; and one offered no explanation. None of the patients in the control group received a personal summary as this was not a feature of that website. Curiously, 3 of these 5 control subjects reported receiving a summary, one of whom reported sharing the summary with his/her provider, one reported not sharing it because it was received at the end of the visit, and one was not sure if he/she shared the summary with the provider.

Half of all subjects (48%) preferred to make the final treatment selection themselves after seriously considering their doctor’s opinion, 13% preferred to share responsibility with their doctor, and 7% preferred their doctor make the final decision after considering their opinion; none preferred to leave all decisions to their provider. Six (35%) of PainAPP users became more engaged with decision making after using the tool, changing their role preferences toward a more collaborative or independent role.

**Fig. 7:**
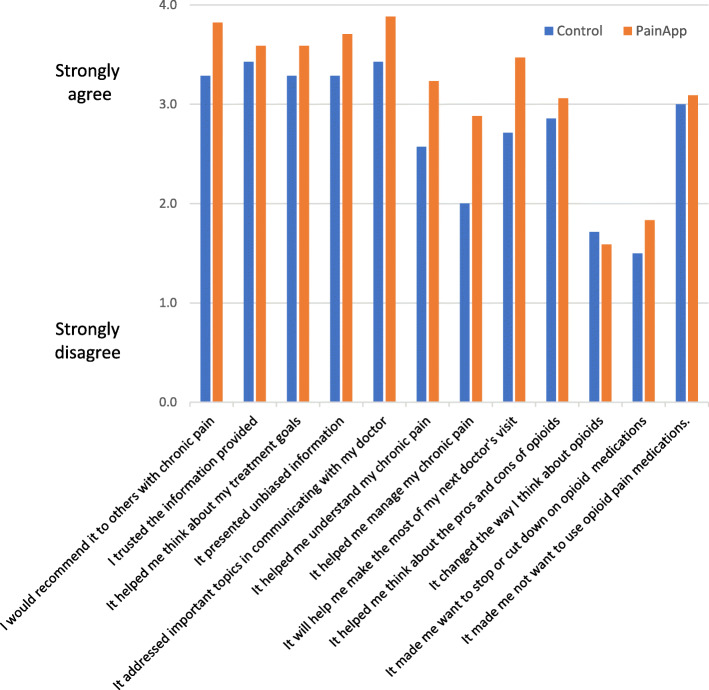
Findings from Beta-testing of PainAPP versus Control (*n* = 24), prior to provider appointment

Both PainAPP and the control website achieved high ratings by most subjects on evaluation measures assessed immediately after use (Fig. 7). Because of the small sample size, high attrition rate, and the small number of people in the control group, no meaningful comparisons can be made. Nearly all PainAPP users strongly or somewhat agreed that *I would recommend it to others with chronic pain* (100%), *It addressed topics that are important in communicating with my doctor (100%)*,and *I trusted the information* (94%).

Statistical testing of the null hypothesis of no difference between the study and controls group could not be done due to small sample size.. Exploratory analyses are shown in Additional file 1: Appendix 1.

We emailed 12 invitations to evaluate the tool to participating providers, which yielded one provider’s evaluations of 2 patients. Both evaluations were non-informative because the two patients evaluated had not chosen to share their report with their provider, leading the provider to respond to all questions with “not applicable”. The providers of patients referred through patient networks could not be contacted.

No harms or unintended effects were reported.

## Discussion

Our formative work confirmed both the challenges and importance of patient-provider communication in managing chronic pain. The most important single treatment goal articulated by patients addressed communication—“having a provider who understand their pain and limitations”. The strategies prioritized by patients to improve patient-provider communication reveal that patients with chronic pain felt neither respected nor trusted by their providers, elements that are essential to effective communication. Strategies prioritized by providers reveal their struggles with gathering information about their patients. While patients focused on transforming providers’ negative attitudes towards patients with chronic pain, providers focused on gathering information about their patients. Providers prioritized collecting biologic over psychosocial data, yet many of the treatment goals identified by patients addressed complex nonbiologic constructs such as preserving independence, pacing oneself, managing emotions, and obtaining affordable treatments. While few of the communication strategies prioritized by patients or providers mapped to key elements of SDM, helping providers with data collection might indirectly promote SDM by freeing up time to discuss the patient’s preferences and concerns.

Preliminary testing suggests that PainAPP was well-received by patients, appears user-friendly, and the summary page appears potentially useful for patients, whether shared with providers or not. Evaluations obtained just after using the tool were generally favorable though findings after the provider visit were mixed. Overall PainAPP appears feasible for use but more rigorous testing is needed.

Our study highlights several challenges with our user-centered design process. Systematically identifying and prioritizing patient and provider goals and communication strategies using cognitive mapping techniques provided valuable insights but was very time consuming. Balancing the priorities of patients with those of providers in the design of the tool was time-consuming and difficult to navigate. Early testers tended to add (rather than remove) sections or features to the tool, resulting in serial lengthening of the tool. Determining the optimal length of the tool was a formidable challenge. After many users found the tool too long, it was redesigned, introduced more skip patterns, emailing content to be read later, and separating it into two parts connected via an emailed link. These changes streamlined the tool but introduced some confusion. Some patients lost the link to Part 2 or did not understand why there was a Part 2.

Tension between comprehensiveness and brevity permeated the development process. Providers requested detailed information about the patient be included in the summary page, yet also wanted the summary to be short and easy to read. Several of the providers who helped guide the development of PainAPP lacked enthusiasm to distribute or use the tool with their patients. This could reflect inadequacies in the summary page content, lack of integration of the tool into clinical workflow, and/ or the heavy workload of providers. The NGT may not have been ideal for identifying implementation strategies for decision support tools because few participating providers had direct experience with these tools. Including providers and practice managers experienced with decision support may have yielded more robust findings.

Beta-testing revealed serious flaws with our study design and the intervention itself that were not previously identified. High attrition rates likely reflected the coupling of a time-intensive study protocol with a time-intensive, two-part online intervention. The opt-in procedures coupled with lengthy consent documents and baseline surveys required nearly as much time as did the intervention itself (21 min versus 30 min, respectively). Most attrition occurred during the intake process, losing 23 (38%) from our invited sample. Attrition was also high during completion of Part 1 of PainAPP (losing an additional 10 participants) and Part 2 of PainApp (losing an additional 3 participants). Intake surveys and assessment evaluations were intentionally administered separated from the intervention to minimize patient burden and help subjects better understand the boundaries of the intervention, but the use of multiple sequential surveys created confusion, as several patients mistook the baseline questionnaire for the intervention itself. Splitting up the intervention into 2 parts compounded this problem. Our choice of control website further compromised our analyses. Whether and how participants used the control site website could not be monitored. Further, many participants were referred to the study through the sponsor of the control website, which may have motivated those subjects to give overly positive evaluations of the control website if randomized to the control group. The functionalities of the two interventions differed substantially--only PainAPP generated a sharable summary and assessed goals--making comparisons of features only included in one website less meaningful. Comparing PainAPP to standard of care might have avoided these difficulties. To minimize patient burden, questions that were asked in PainAPP (but not the control) were not duplicated in the baseline survey for those assigned to PainAPP. However, this compromised our pre-post evaluation of PainAPP because these items were effectively asked after completing parts of the intervention, thus not capturing pre-intervention status. PainAPP may have altered participants’ perspective and attitudes when answering questions, given that we observed a shift in preferences for decision-making after viewing the intervention. A retrospective pre-post design may have been more appropriate for this intervention. Such a design is more sensitive and valid [69] than a randomized controlled trial if the intervention prompts a change in the frame of reference that participants use to assess their attitudes, underestimating the intervention’s effects [70–74].

Embedding PainAPP into the electronic health record (EHR) should facilitate its integration into clinical workflow and minimize many of the difficulties encountered. Dissemination could be triggered when scheduling appointments, and the summary page could be automatically embedded into the patient’s record, facilitating sharing at point-of-care. With additional resources, PainAPP could potentially be embedded into EHRs, either in its entirely or in modules. It could also be customized to different clinical practices or settings and linked to quality improvement metrics. PainAPP could also be reprogrammed as a mobile App. However, reprogramming an interactive tool as complex as PainAPP into either an EHR or mobile App would be a daunting and error-prone undertaking. To integrate PainAPP into clinical workflow without the benefit of being integrated into an EHR, PainAPP (or an abbreviated version of PainApp) should be distributed to patients 1–2 weeks before their appointments and delivery of their summary report to their provider should be automated. The PainAPP summary could replace the cumbersome paper-based medical history form that is often used in waiting rooms.

Our beta-testing provided insights to guide future evaluation and distribution. Referrals from both providers and patient-networks appear feasible and capture different population segments. Referrals from pain specialists exclude those lacking access to specialty care; referrals from patient networks exclude socially isolated patients. Combined approaches for dissemination, using both provider and patient networks, may be the optimum strategy for broad distribution.

Our study has many limitations. Our sample size for NGTs and cognitive mapping was small. However, we were able to achieve model convergence with non-parametric multidimensional scaling and hierarchical cluster analysis. Two references support the use of these methods where the power analysis was carried out via Monte Carlo simulation with small sample size equivalent to the magnitude of our study’s sample size. One demonstrates sufficient power for a general multidimensional scaling model with the number of clusters up to 4 [75]. Another examines power issues for hierarchical cluster models in the correct identification of the “true” number of partitions and the cluster size and also used simulation study on a small sample size dataset [76].To improve the representativeness of the sample, we recruited diverse participants from across the U.S. Nonetheless, our small sample size limits the generalizability of our findings. Our sample appeared representative with regard to age and gender, though less so with respect to race [77]. Our pilot study findings relied on self-report, which may have led to a tendency for more favorable responses. We did not confirm a diagnosis of chronic pain, but our referral sources made incorrect diagnoses unlikely. The tool is designed to be used online or on a mobile device, thus restricting access for some patients but expanding reach to others. Attempts to obtain provider evaluations of the patient encounter were unproductive. Survey invitations emailed from the study’s secure Qualtrics server were frequently blocked from providers’ email servers by institutional firewalls, going into spam folders. When firewalls could eventually be bypassed, providers were confused when they received multiple emails about patients they had seen too long ago to remember details of the encounter. Some providers did not understand that they were expected to complete evaluations even if the patient did not share the summary page or was in the control group. Further, we had no contact information for providers of participants referred through patient networks. Addressing firewall barriers prior to study initiation and improving instructions about completing provider surveys may have helped, as would integration into the EHR.

There appears to be an unmet need of patient-centered tools to help manage chronic pain. Decision aids that have been evaluated for chronic musculoskeletal pain are less effective than those developed for other conditions. Furthermore, a public directory of decision aids does not list any available decision aids targeting chronic pain [78]. However, a growing number of mobile pain management Apps are becoming available. A recent review [79] identified 36 mobile pain management Apps, most serving as pain diary tools. However, most of these apps (69%) did not involve clinicians in App development, none systematically engaged patients with chronic pain in development, and none were considered to be suitable for clinical usage due to lack of HIPAA compliance. That review concluded that there are no pain management Apps designed for clinical use by physicians.

Given the urgency for deploying new approaches to tackle the ongoing opioid epidemic, PainAPP has been embedded into a website (ThePainAPP.com, Additional file 2: Appendix 2) that does not require provider or patient referral, removing the need for prescreening or disclosure of personal identifiers. To avoid the challenges introduces by using a linear, sequential design, patients and providers can choose how they would like to learn— browsing by topic, using only the Patient Engagement/Education tool, the Comprehensive Pain Assessment tool, or both.

Misunderstanding patient treatment preferences is widespread and can result in unnecessary or even harmful treatments [80]. Improving the lives of patients with chronic pain requires that patients understand their choices and the consequences of those choices, and that providers understand their patients, which requires good communication between the two. This manuscript illustrates the value of conducting beta-testing of SDM tools in real-world settings before large-scale clinical testing, consistent with other recommendations [51, 81]. This study identifies the treatment goals of patients with chronic pain as well as communication strategies to improve chronic pain discussions from the perspective of both patients and providers. These findings, coupled with lessons learned about designing and testing patient- and clinician-centered tools, may provide useful insights for other tool developers. PainAPP appears to be feasible for use at this time, though further adaptation and testing is needed to assess its impact on patients and providers.

## Supplementary information

**Additional file 1: Appendix 1.** Exploratory Analyses of Post-provider Visit in each Study Group.

**Additional file 2: Appendix 2.** Screen Shots from ThePainAPP.com (hosts the PainAPP tool).

## Data Availability

The datasets used and/or analyzed during the current study are available from the corresponding author on reasonable request.

## Footnotes

1 This response was: “We discuss my fibromyalgia pain and changes each appointment briefly”
