# Supplementary material for: Improving patient-provider communication about chronic pain: development and feasibility testing of a shared decision-making tool
Source: BMC Med Inform Decis Mak. 2020 Oct 17;20:267. doi: 10.1186/s12911-020-01279-8 (PMC7568350; doi:10.1186/s12911-020-01279-8)
Supplement: Supplementary file 1 — Additional file 1: Appendix 1. Exploratory Analyses of Post-provider Visit in each Study Group. [file 12911_2020_1279_MOESM1_ESM.docx]

**Appendix 1.** **Exploratory Analyses of Post-provider Visit in each Study Group**

| **Outcome Measure** | **Study** | | | | **Control** | | | |
| --- | --- | --- | --- | --- | --- | --- | --- | --- |
|  | n | Median | Range (min, max) | IQR* | n | Median | Range (min, max) | IQR |
| Current pain | 17 | 6 | 7 (2, 9) | 3.00 | 5 | 5 | 4 (4, 8) | 3.00 |
| Pain Interference | 17 | 13 | 19 (6, 25) | 8.50 | 5 | 16 | 10 (9,19) | 7.50 |
| CAHPS** | 16 | 29 | 22 (13,35) | 11.25 | 5 | 31 | 14 (20,34) | 8.00 |
| COMRADE: Satisfaction with Communication | 15 | 19 | 13 (7,20) | 9.00 | 2 | 18 | 4 (16,20) | -- |
| Confidence that treatment plan is working | 17 | 2 | 3 (0,3) | 2.00 | 5 | 1 | 3 (0,3) | 3.00 |
| Current Opioid use | 17 | 0 | 1 (0,1) | 1.00 | 4 | 1 | 1 (0,1) | 1.00 |
| Involvement in decision- making to the extent desired | 17 | 1 | 1 (0,1) | 0.00 | 5 | 1 | 1 (0, 10) | 1.00 |

*IQR: Interquartile range.

**CAHPS: Consumer Assessment of Healthcare Providers and Systems.
