# Supplementary figures and images for: Improving patient-provider communication about chronic pain: development and feasibility testing of a shared decision-making tool
Source: BMC Med Inform Decis Mak. 2020 Oct 17;20:267. doi: 10.1186/s12911-020-01279-8 (PMC7568350; doi:10.1186/s12911-020-01279-8)

**Appendix 2. Screen Shots from ThePainAPP.com (hosts the PainAPP tool)**

**
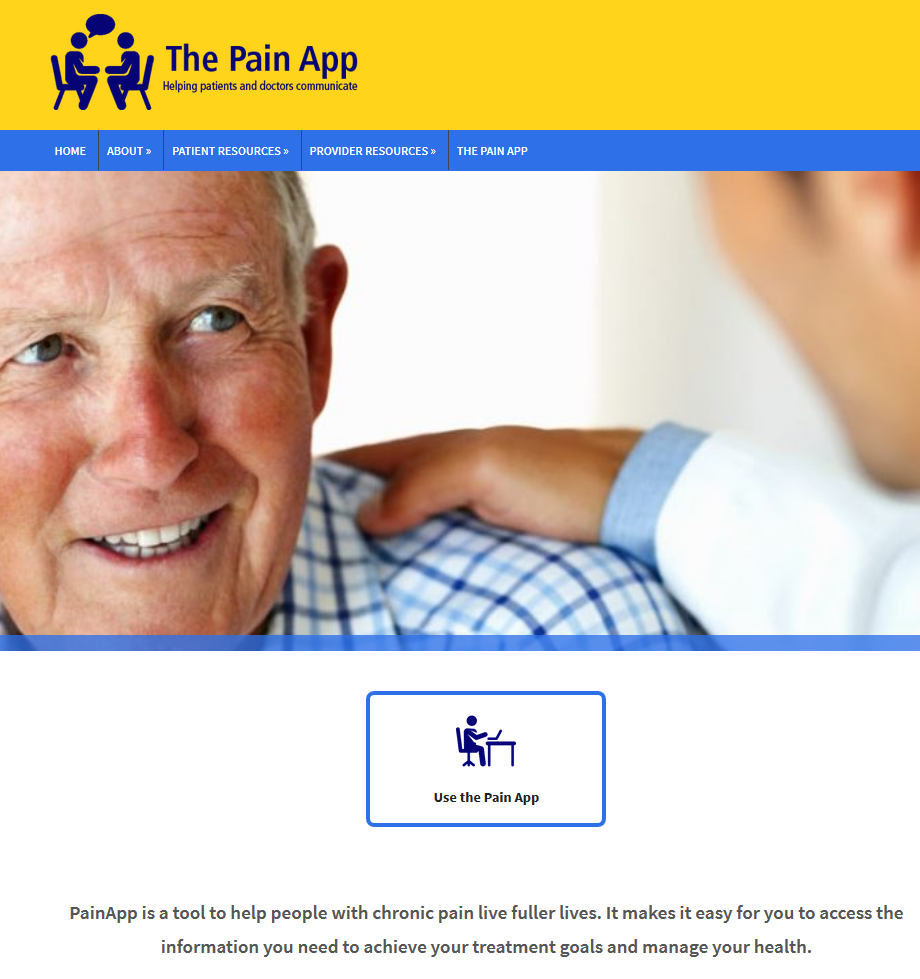
**


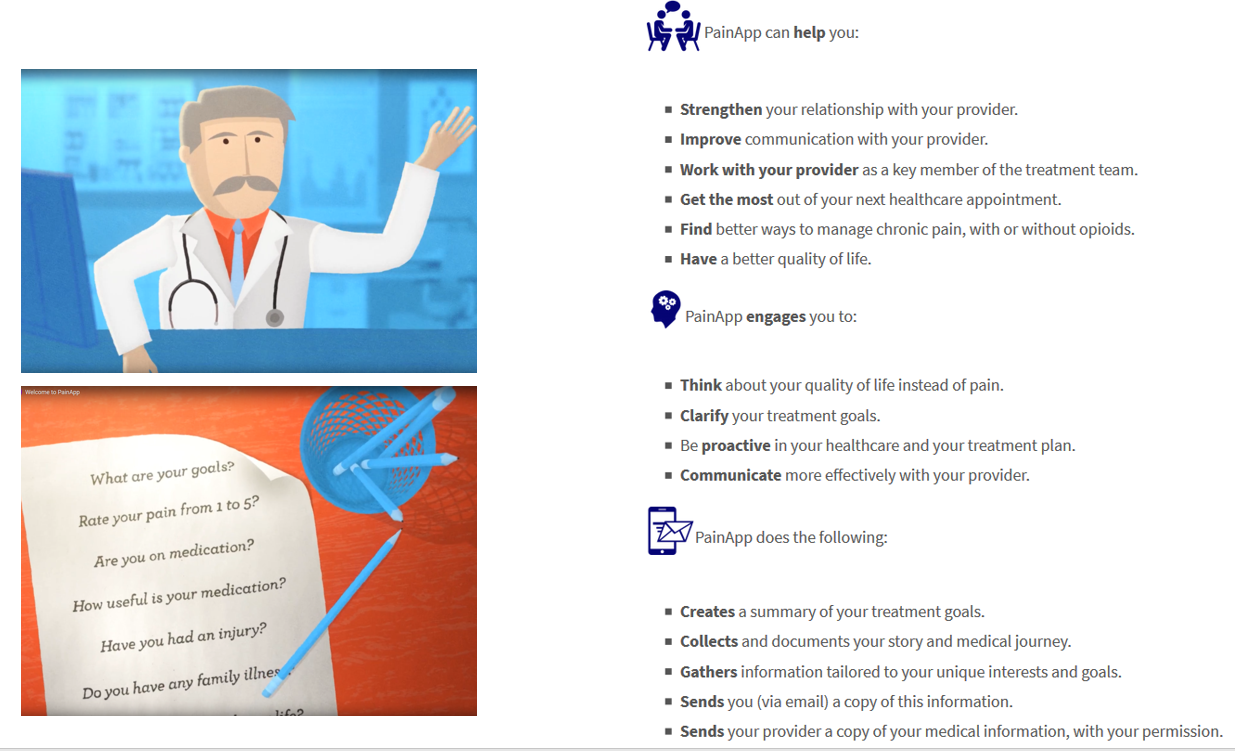

Supplement: Supplementary file 2 — Additional file 2: Appendix 2. Screen Shots from ThePainAPP.com (hosts the PainAPP tool). [file 12911_2020_1279_MOESM2_ESM.docx]
